# Influence of cigarette smoking on biventricular systolic function independent of respiratory function: a cross-sectional study

**DOI:** 10.1186/s12872-020-01732-6

**Published:** 2020-10-15

**Authors:** Yusuke Watanabe, Kazuko Tajiri, Atsuko Suzuki, Hiroyuki Nagata, Masayuki Kojima

**Affiliations:** 1 Department of Internal Medicine, Hitachiomiya Saiseikai Hospital, 3033-3 Tagouchichou, Hitachiomiya, Ibaraki 319-2601 Japan; 2 Department of Cardiology, Faculty of Medicine, University of Tsukuba, Tsukuba, Japan; 3 Department of Clinical Laboratory, Hitachiomiya Saiseikai Hospital, Hitachiomiya, Japan; 4 Department of Surgery, Hitachiomiya Saiseikai Hospital, Hitachiomiya, Japan

**Keywords:** Cigarette smoking exposure, Echocardiography, Cardiac systolic function, Spirometry

## Abstract

**Background:**

Cigarette smoking harms nearly every organ, including the heart and lungs. A comprehensive assessment of both cardiac and respiratory function is necessary for evaluating the direct effects of tobacco on the heart. However, few previous studies examining the effects of cigarette smoking on cardiac function included an assessment of lung function. This cross-sectional study investigated the influence of cigarette smoking on cardiac function, independent of respiratory function.

**Methods:**

We retrospectively reviewed the medical records of 184 consecutive cases that underwent both spirometry and transthoracic echocardiography around the same time (within 1 month) in one hospital from April 2019 to March 2020. Participants were classified into three groups based on lifetime smoking exposure (pack-years): non-smoker (n = 49), low exposure (1–20 pack-years, n = 40), and high exposure (≥ 20 pack years, n = 95). Multiple linear regression analysis was used to assess the relationship among cigarette smoking, and cardiac and respiratory functions. The relationship between selected dependent variables and lifetime pack-years was assessed in two models with multiple linear regression analysis. Model 1 was adjusted for age and male sex; and Model 2 was adjusted for Model 1 plus forced expiratory volume percentage in 1 s and forced vital capacity percentage.

**Results:**

Compared with the non-smokers, the participants with high smoking exposure had lower left ventricular (LV) systolic function and larger LV size. Multiple linear regression analysis revealed a negative association of cumulative lifetime pack-years with LV and right ventricular (RV) systolic functions, even after adjustment for age, sex, and spirometric parameters (forced expiratory volume percentage in 1 s and forced vital capacity percentage). Meanwhile, there was no significant association of smoking exposure with LV diastolic function (E/e′ and E/A) and RV diastolic function (e′t and e′t/a′t).

**Conclusions:**

Cumulative smoking exposure was associated with a negative effect on biventricular systolic function in patients with relatively preserved cardiac function, independent of respiratory function.

## Background

Cigarette smoking is well established as a major cause of cardiovascular disease [1–3]. Smoking is not only a risk factor for coronary artery disease, but also for heart failure [4]. Although several studies have evaluated the relationship between cigarette smoking and cardiac function, their results were inconsistent [5, 6]. Nonetheless, cigarette smoking has a known negative effect on lung function [7, 8]. There is a close relationship between lung and heart function. In addition, even subclinical impairment of respiratory function has been reported to be associated with cardiac dysfunction [9]. A comprehensive assessment of both cardiac and respiratory function is important for investigating the direct effects of tobacco use on the heart. However, few previous studies examining the effects of cigarette smoking on cardiac function included an assessment of lung function. Therefore, we investigated the cross-sectional association between cigarette smoking and cardiac function independent of respiratory function in individuals who underwent simultaneous echocardiography and spirometry.

## Methods

This single-hospital retrospective observational study was conducted at Hitachiomiya Saiseikai Hospital. We reviewed the medical records of all consecutive cases that underwent both spirometry and transthoracic echocardiography around the same time (within one month) in our hospital from April 2019 to March 2020. The exclusion criteria were: (1) severe valvular disease (defined and diagnosed according to the guidelines of the American Society of Echocardiography [10–12]) or a history of valvular surgery or cardiac surgery, (2) a clinically evident history of lung disease and/or an abnormal finding of the lung field on imaging, (3) a history of ischemic heart disease (i.e., acute myocardial infarction and/or prevalence of local wall abnormality), (4) a participant with acute disease at examination by echocardiography and/or spirometry, (5) atrial fibrillation at examination with electrocardiogram, and (6) left ventricular ejection fraction (LVEF) ≤ 40%. This study was approved by our Institutional Review Board (ID 20-02), and it was carried out in accordance with the Declaration of Helsinki for experiments involving humans. The requirement for written informed consent was waived due to the retrospective nature of the study by our Institutional Review Board.

In echocardiography, data acquisition was performed by one of the expert sonographers using an EPICcv (Philips Medical Systems, Andover, MA, USA), equipped with a 5-MHz transducer, according to the established guidelines [10–15]. Echocardiography was generally performed without acute smoking exposure. Echocardiographically measured and derived variables were determined as follows: left ventricular mass (LVM) was calculated from left ventricular (LV) chamber size and wall thickness dimensions; 2-dimentional LVEF was computed from LV end-diastolic and end-systolic volumes which were calculated according to the bi-plane disk summation method in apical four- and two-chamber views; pulsed-wave Doppler was performed with the sample volume placed at the level of the mitral leaflet tips to obtain peak early (E) and late (A) diastolic transmitral inflow velocities and E-wave deceleration time; and tissue Doppler was performed to acquire mitral early (e′) and late (a′) diastolic annular velocities on the lateral side of the wall. To assess LV diastolic function, we used E/A ratio and E/e′ ratio. Right ventricular (RV) end-diastolic area was measured from an RV-focused apical four-chamber view. Right ventricular fractional area change (RVFAC), tricuspid annular plane systolic excursion (TAPSE), and RV peak systolic tissue Doppler velocity captured different dimensions of RV systolic function. RVFAC was obtained by tracing the RV endocardium from the annulus, along the free wall to the apex, back to the apex, then along the interventricular septum. To evaluate the RV diastolic function, tissue Doppler parameters were used: early diastolic peak velocity (e′t), late diastolic peak velocity (a′t) of the tricuspid annulus, and its ratio (e′t/a′t). Strain analyses were performed using a semi-automated speckle tracking technique (aCMQ, QLAB Cardiac Analysis, Philips Medical Systems, Andover, MA, USA). Three apical views were used to obtain an average global peak systolic longitudinal strain (GLS) with systole (Additional file 1: Figure 1).

The patients’ medical records were carefully reviewed in order to obtain information about their smoking history. In the instances where information on smoking status was lacking, they were interviewed directly. Cumulative smoking exposure was determined in terms of pack-years by multiplying the number of years the participant has smoked by the average number of packs per day [16]. We divided the participants into three groups according to the cumulative lifetime smoking exposure: non-smoker group including the participants who had never smoked a cigarette or other tobacco products before, low exposure group (1–20 pack-years), and high exposure group (≥ 20 pack years) [17]. Hypertension was defined as using medical treatment for hypertension and/or a history of hypertension before admission. Dyslipidemia was defined as triglyceride level ≥ 150 mg/dl, low-density lipoprotein cholesterol level ≥ 140 mg/dl, high-density lipoprotein cholesterol level ≤ 40 mg/dl, using medical treatment for dyslipidemia, or a history of dyslipidemia. Diabetes mellitus was defined as hemoglobin A1c level ≥ 6.5% (as National Glycohemoglobin Standardization Program value), using medical treatment for diabetes mellitus, or a history of diabetes mellitus. We calculated the estimated glomerular filtration rate (eGFR) from serum creatinine levels, age, weight, and gender using the following formula; eGFR (ml/min/1.73 m^2^) = 194 × s-Cr (− 1.094) × age (− 0.287) × 0.739 (if female) [18].^.^ Chronic obstructive pulmonary disease (COPD) was defined as post-bronchodilator forced expiratory volume in 1 s (FEV1)/forced vital capacity (FVC) < 0.70, which confirms the presence of persistent airflow limitation on spirometry or a history of medication [19].

### Methods for measurement of spirometric parameters

The Berglund equation was used to predict FEV1.0 according to the following calculations: FEV1.0 (L) in males = 3.44 × height (m) – 0.033 × age − 1; FEV1.0 (L) in females = 2.67 × height (m) − 0.027 × age − 0.5 [20, 21]. The forced expiratory volume percentage in one second (FEV1.0%) = measured FEV1.0/measured FVC. The percent predicted forced expiratory volume in one second (%FEV1.0) = measured FEV1.0/predicted FEV1.0. The Baldwin equation was used to predict VC and FVC as follows: VC (L) in males = (27.63 – 0.0112 × age) × height (m); VC (L) in females = (21.78 – 0.101 × age) × height (m). The vital capacity percentage = measured VC/predicted VC [20, 21]. The percent predicted forced vital capacity = measured FVC/predicted FVC. Spirometry was performed with MICROSPIRO HI-80 (NIHON KODEN, JAPAN).

### Statistical analysis

The normality was tested with the Shapiro–Wilk test. Continuous data are expressed as mean ± standard deviation (SD) for normally distributed variables and median [25th–75th percentile] for non-normally distributed variables. Normally distributed continuous variables were compared between the groups using a one-way analysis of variance (ANOVA) with a post hoc analysis with Tukey test or Games–Howell test. Non-normally distributed continuous variables were compared using a Kruskal–Wallis test with a post hoc analysis with Dann-Bonferroni method. Categorical variables are expressed as numbers (percentage) and were compared using Pearson’s χ^2^ test or Fisher’s exact test.

Multiple linear regression analysis was used to assess the relationship among cigarette smoking, and cardiac and respiratory functions. Dependent variables were selected from variables that could indicate the ventricular systolic and diastolic function on echocardiography as follows: LVEF, E/e′, E/A, RVFAC, e′t, and e′t/a′t. We assessed the relationship between the selected dependent variables and the lifetime pack-years in two models with a multiple linear regression analysis. First, Model 1 was adjusted for age and male sex; and Model 2 was adjusted for Model 1 plus FEV1.0% and forced vital capacity percentage (%FVC). Age and sex were selected as independent variables because they were well-known parameters which could affect the cardiac function. Meanwhile, FEV1.0% and %FVC were selected as independent variables because they were important parameters on spirometry that could clearly indicate respiratory condition [22]. Beta coefficients were interpreted as the absolute change in dependent variables per change in the pack-years intensity. A *p* value < 0.05 was considered statistically significant. All analyses were performed using statistical software, SPSS 26.0/Windows (SPSS, Chicago, IL).

## Results

The final study population included 184 subjects (69.6% men, mean age: 70.1 ± 13.3 years old), divided into the following three groups: non-smoker group (n = 49), low smoking exposure group (n = 40), and high smoking exposure group (n = 95). Table 1 shows a comparison of clinical characteristics among the three groups. Non-smokers were significantly older, more likely to be women, and had higher cardio-thoracic ratio compared with the other two groups. The individuals with high smoking exposure were older and more likely to be men compared with those in the low exposure group. Meanwhile, body mass index was comparable among the three groups and within normal range.

**Table 1:** Clinical characteristics of participants according to smoking exposure

	Cumulative smoking exposure			
	Non-smoker (n = 49)	Low exposure (n = 40)	High exposure (n = 95)	*p* value
Age (years old)	78.0 (69.5–83.0)	65.5 (51.0–75.5)*	70.0 (64.5–81.0)*^,†^	< 0.001
Male sex	12 (24.5)	29 (72.5)	87 (91.6)	< 0.001
Body mass index (kg/m^2^)	23.2 (21.6– 25.5)	23.9 (21.4–25.8]	23.2 (20.7–25.5)	0.72
Systolic blood pressure (mmHg)	139 (126–155)	135 (125–146)	136 (124–146)	0.36
Diastolic blood pressure (mmHg)	74 ± 12	77 ± 13	77 ± 13	0.42
Heart rate (beats/min)	71 ± 9	66 ± 10	68 ± 12	0.21
Hypertension	33 (67.3)	20 (50.0)	47 (49.5)	0.10
Dyslipidemia	28 (57.1)	24 (60.0)	53 (55.8)	0.90
Diabetes mellitus	7 (14.3)	13 (32.5)	25 (26.3)	0.11
Estimated glomerular filtration rate (ml/min/1.73 m^2^)	64.0 (51.0–75.0)	66.5 (52.0–80.0)	63.5 (54.0–72.0)	0.50
Chronic obstructive pulmonary disease	0 (0.0)	1 (2.5)	10 (10.5)	0.017
Bronchial asthma	3 (6.1)	2 (5.0)	1 (1.1)	0.14
Current smoke	0 (0.0)	6 (15.0)	27 (28.4)	< 0.001
Pack-years	–	10 (6–15)*	40 (30–50)*^,†^	< 0.001
Smoking exposure duration (years)	–	15 (10–20)*	40 (30–47)*^,†^	< 0.001
Cardio-thoracic ratio (%)	51.2 ± 5.4	46.2 ± 4.1*	46.7 ± 3.7*	< 0.001
The reason for spirometry and echocardiography				0.071
Symptomatic (chest pain, cough, and shortness of breath) (%)	7 (14.3)	11 (27.5)	23 (24.2)	
Asymptomatic and/or periodical medical examination (%)	27 (55.1)	25 (62.5)	59 (62.1)	
Pre-operation examination without symptom (%)	15 (30.6)	4 (10.0)	13 (13.7)	
Medication				
ACE-I and/or ARB	18 (36.7)	10 (25.0)	30 (31.6)	0.51
Calcium channel blockers	19 (38.8)	11 (27.5)	32 (33.7)	0.54
β-blockers	5 (10.2)	2 (5.0)	7 (7.4)	0.67
Diuretics	5 (10.2)	1 (2.5)	8 (8.4)	0.38
Statins	13 (26.5)	11 (27.5)	25 (26.3)	1.00
Long-acting muscarinic antagonists	0 (0.0)	0 (0.0)	6 (6.3)	0.067
Long-acting β2-antagonists	2 (4.1)	1 (2.5)	6 (6.3)	0.74
Inhaled corticosteroids	2 (4.1)	1 (2.5)	2 (2.1)	0.84

Categorical variables are indicated as number and (%). Continuous variables are indicated as mean ± SD for normally distributed variables and median [25th–75th percentile] for non-normally distributed variables

ACE-I: angiotensin-converting enzyme inhibitor, ARB: angiotensin receptor blocker

^*^p value < 0.05 for versus non-smoker

^†^*p* value < 0.05 for versus low exposure

Table 2 shows the comparison of cardiac function by echocardiography among the three groups. When compared with non-smokers, the participants with high smoking exposure had lower LVEF; larger LV dimension and LV volume; slower A wave velocity; higher E/A; larger RV area and tricuspid valve annular diameter; and higher e′t/a′t. The participants with low smoking exposure had larger LV dimension and LV volume; slower A wave velocity; higher E/A, larger RV area; higher e′t/a′t; and a larger inferior vena cava. There were no significant differences in echocardiographic parameters between the low and high exposure groups. The comparison of respiratory function by spirometry among the three groups is shown in Additional file 2: Table 1. Overall, non-smokers had better respiratory function than the participants with any smoking exposure. The degree of FEV1.0, FVC, and vital capacity (VC) were significantly lower in the non-smoker group than in the smoking exposure groups. Meanwhile, the degree of FEV1.0% and %FEV1.0 were significantly better in the non-smoker group than in the smoking exposure groups.

**Table 2:** Echocardiographic characteristics of participants according to smoking exposure

	Cumulative smoking exposure			
	Non-smoker (n = 49)	Low exposure (n = 40)	High exposure (n = 95)	*p* value
Left ventricular ejection fraction (%)	70.4 (64.7– 72.2)	67.3 (62.6–70.9)	65.3 (62.5–69.3)*	0.001
Interventricular septum thickness (mm)	9.2 ± 1.2	9.1 ± 1.0	9.4 ± 1.2	0.31
Left ventricular end-diastolic diameter (mm)	41.0 (37.1–44.2)	45.1 (41.9–47.7)*	44.5 (41.5–47.6)*	0.002
Left ventricular end-systolic diameter (mm)	24.6 (22.3–25.8)	26.7 (25.1–30.0)*	28.1 (25.6–30.4)*	< 0.001
Posterior left ventricular wall thickness (mm)	9.3 (8.5–10.1)	9.5 (8.8–10.0)	9.7 (8.8–10.2)	0.31
Left atrial diameter (mm)	33.8 ± 4.9	34.4 ± 4.8	34.0 ± 5.4	0.84
Left atrial volume index (ml/m^2^)	32.0 (25.5– 39.6)	28.7 (24.1–35.4)	27.8 (24.7–33.3)	0.076
Left ventricular end-diastolic volume index (ml/m^2^)	39.4 (34.0–50.7)	48.6 (41.1–55.7)*	46.1 (38.7–53.9)*	0.013
Left ventricular end-systolic volume index (ml/m^2^)	12.2 (10.1–14.8)	14.9 (13.3–19.3)*	16.1 (13.1–18.9)*	< 0.001
Left ventricular mass index (g/m^2^)	80.0 (67.7–92.3)	82.9 (70.6–90.7)	82.9 (71.7–94.9)	0.51
E wave velocity (cm/s)	62.3 ± 16.0	61.8 ± 11.9	61.0 ± 15.7	0.87
E wave deceleration time (ms)	204 (180–232)	192 (168–243)	197 (162–259)	0.80
A wave velocity (cm/s)	85.5 (71.0–100)	73.4 (61.9–84.1)*	75.8 (61.5–88.7)*	0.003
E/A	0.70 (0.60–0.80)	0.85 (0.70–1.00)*	0.80 (0.60–1.00)*	0.020
e′ velocity (cm/s)	7.2 (6.0–8.3)	8.4 (6.6–10.6)*	8.0 (6.7–9.8)	0.004
a′ velocity (cm/s)	9.4 (8.0–11.0)	9.9 (8.1–11.4)	9.4 (8.2–11.5)	0.99
E/e′ (lateral)	7.6 (6.2–11.0)	7.6 (6.6–8.9)	7.6 (5.8–8.9)	0.29
Left ventricular global longitudinal strain (%)	− 20.6 ± 1.6	− 20.3 ± 1.3	− 20.2 ± 1.8	0.37
Right atrial area index (cm/m^2^)	8.8 (7.6–10.3)	8.2 (7.4–9.1)	8.4 (7.1–10.3)	0.58
Right ventricular end-diastolic area index (cm^2^/m^2^)	8.0 (7.1–9.6)	8.8 (7.2–9.8)	8.9 (7.6–10.4)	0.078
Right ventricular end-systolic area index (cm^2^/m^2^)	4.0 (3.7–4.9)	4.8 (4.0–6.0)*	4.9 (4.1–5.9)*	0.001
Right ventricular fractional area change (%)	48.4 (42.4–54.5)	46.0 (40.7–50.5)	45.7 (40.9–49.2)	0.063
Tricuspid valve annular diameter (cm)	26.8 ± 4.7	28.4 ± 4.5	30.0 ± 4.4*	< 0.001
Tricuspid annular plane systolic excursion (mm)	20.6 ± 4.5	20.6 ± 3.2	21.4 ± 3.9	0.40
s't velocity (cm/s)	11.2 (10.3–13.3)	11.5 (10.2–12.9)	11.9 (10.7–13.3)	0.72
e't velocity (cm/s)	7.8 (6.3–9.4)	8.3 (7.3–10.6)	8.2 (6.9–9.6)	0.14
e't/a't	0.55 (0.43–0.64)	0.68 (0.54–0.85)*	0.63 (0.49–0.76)*	0.003
Right ventricular global longitudinal strain (%)	− 21.2 ± 2.8	− 20.9 ± 2.1	− 21.1 ± 2.8	0.88
Inferior vena cava (mm)	12.8 (10.9–14.3)	14.5 (12.5–15.6)*	13.6 (12.0–15.7)	0.034

Variables are indicated as mean ± SD for normally distributed variables and median [25th–75th percentile] for non-normally distributed variables

A, late diastolic transmitral inflow peak velocity; a′t, late diastolic peak velocity of the tricuspid annulus; E, early diastolic transmitral inflow peak velocity; e′, mitral early diastolic annular peak velocity; e′t, early diastolic peak velocity of the tricuspid annulus; s′t, systolic wall motion velocity of the tricuspid annulus

^*^p value < 0.05 for versus non-smoker

^†^*p* value < 0.05 for versus light smoker

Table 3 presents the association between lifetime pack-years and cardiac function. The LVEF, RVFAC, and parameters of ventricular systolic function were negatively correlated with the number of lifetime pack-years. The LV end-systolic volume index, RV end-systolic area index, and parameters of end-systolic ventricular volume were positively associated with the pack-years intensity. The E/A, E/e′, e′t, e′t/a′t, and parameters of ventricular diastolic function were not associated with the number of pack-years. The respiratory parameter regarding airflow limitation, such as FEV1.0% and %FEV1.0, were negatively associated with the increasing number of lifetime pack-years (Additional file 2: Table 2).

**Table 3:** Correlation between lifetime pack-years and echocardiographic parameters

Variable	Correlation coefficient	*p* value
Left ventricular ejection fraction (%)	− 0.248	0.001
Left ventricular end-diastolic diameter (mm)	0.182	0.013
Left ventricular end-systolic diameter (mm)	0.281	< 0.001
Left ventricular end-diastolic volume index (ml/m^2^)	0.108	0.15
Left ventricular end-systolic volume index (ml/m^2^)	0.255	< 0.001
Left ventricular mass index (g/m^2^)	0.063	0.39
Left atrial volume index (ml/m^2^)	− 0.116	0.12
A velocity (cm/s)	− 0.136	0.066
E/A	0.046	0.54
E/e′	− 0.062	0.41
Right ventricular end-diastolic area index (cm^2^/m^2^)	0.156	0.037
Right ventricular end-systolic area index (cm^2^/m^2^)	0.28	< 0.001
Right ventricular fractional area change (%)	− 0.173	0.021
Tricuspid valve annular diameter (mm)	0.273	< 0.001
e′t velocity (cm/s)	0.032	0.67
e′t/a′t	0.087	0.25
Inferior vena cava (mm)	0.109	0.14

A, late diastolic transmitral inflow peak velocity; a′t, late diastolic peak velocity of the tricuspid annulus; E, early diastolic transmitral inflow peak velocity; e′, mitral early diastolic annular peak velocity; e′t, early diastolic peak velocity of the tricuspid annulus

Table 4 shows the effect of lifetime pack-years on ventricular systolic and diastolic function. There was a negative association between cumulative lifetime pack-years and left and right ventricular systolic functions (LVEF and RVFAC, respectively), even after adjustment for age, sex, FEV1.0%, and %FVC (Table 4); this suggests that cigarette smoking could cause biventricular systolic dysfunction independent of respiratory function. Meanwhile, there was no significant association between the lifetime pack-years and LV diastolic function (E/e′ and E/A), RV diastolic function (e′t and e′t/a′t), or other echocardiographic parameters, except for tricuspid valve annular diameter (Table 4 and Additional file 2: Table 3).

**Table 4:** Effects of lifetime pack-years on ventricular systolic and diastolic function

	Model 1		Model 2	
	β coefficient	*p* value	β coefficient	*p* value
Left ventricular ejection fraction (%)	− 0.048 (− 0.087, − 0.009)	0.016	− 0.047 (− 0.088, − 0.006)	0.026
E/e'	− 0.007 (− 0.028, 0.014)	0.52	− 0.004 (− 0.026, 0.018)	0.74
E/A	− 0.00003 (− 0.002, 0.002)	0.97	− 0.0002 (− 0.002, 0.002)	0.87
Right ventricular fractional area change, %	− 0.074 (− 0.136, − 0.001)	0.020	− 0.071 (− 0.137, − 0.005)	0.035
e't velocity, cm/s	− 0.009 (− 0.026, 0.009)	0.35	− 0.011 (− 0.030, 0.008)	0.26
e't/a't	− 0.0004 (− 0.002, 0.001)	0.59	− 0.001 (− 0.003, 0.001)	0.22

Beta coefficients were interpreted as the absolute change in the dependent variable per change in the intensity of pack year

Model 1 is adjusted for age and sex. Model 2 is adjusted for Model 1 plus forced expiratory volume percentage in one second (FEV1.0%) and percent predicted forced vital capacity (%FVC)

A, Late diastolic transmitral inflow peak velocity; a′t, Late diastolic peak velocity of the tricuspid annulus; E, Early diastolic transmitral inflow peak velocity; e′, Mitral early diastolic annular peak velocity; e′t, Early diastolic peak velocity of the tricuspid annulus

## Discussion

There were two main findings of the present study: (1) the individuals with high smoking exposure had lower LV systolic function and larger LV size; and (2) the dose of lifetime cumulative smoking exposure was significantly associated with worse LV and RV systolic function independent of respiratory function. These findings reveal that cigarette smoking exposure may result in the development of cardiac dysfunction in a direct and continuous dose-dependent fashion and thus, may contribute to the heart failure epidemic.

There were several previous studies regarding the relationship between smoking exposure and worsening cardiac function [1, 6, 23–25]. Specifically, some studies reported that LV mass index could increase with higher exposure to smoking [5, 6]. However, in the present study, the lifetime smoking exposure had no significant association with LV mass index. Meanwhile, one previous study showed that cigarette smoking was closely related to low LV mass index [26]. One explanation was that LV mass index was reported to have an association with several factors including age, body weight, height, physical activity, and blood pressure [26]. The biased effect of the participants’ background in the present study might have affected the relationship between smoking exposure and LV mass index. In particular, the patients in the present study had evidently lower BMI compared with the previous studies [5, 6], which might have affected the ventricular morphology including LV mass index. Therefore, the result of LV mass index should also be carefully interpreted taking the participants’ background into consideration. Furthermore, the implication of the present study was to investigate the association between smoking exposure and cardiac function while considering respiratory function. Although the effects of respiratory function against cardiac function have already been reported [27–29], there were few studies investigating the relationship between smoking exposure and cardiac function, after adjusting for respiratory function. In the present study, the higher the participants’ smoking exposure, the larger the values of FVC and VC. Conversely, the cardio-thoracic ratio of the patients with smoking exposure was lower than that of the non-smoker patients, which could suggest the impact of the effects of smoking exposure on lung expansion. A previous study also reported that patients with severe emphysema had significantly lower stroke volume index and LV end-diastolic area index [30]. Although the interaction between heart and lung was observed (even in the present study), the present study showed that cumulative smoking exposure could be significantly related to worse LV and RV systolic function independent of respiratory function.

Cigarette smoking could have serious effects on the systemic function, especially cardiac and respiratory functions. Several mechanisms regarding smoking exposure and cardiovascular dysfunction have been suggested previously. In animal models, cigarette smoking has been shown to induce ventricular hypertrophy and remodeling with increased serum levels of norepinephrine [6]. Continuous exposure to smoking could stimulate the autonomic nervous system and induce cardiac remodeling [6]. These injury models would have effects on the RV function as well as the LV function [6]. Furthermore, cigarette smoking could cause subclinical cardiovascular damage [31]. This damage could be associated with increased blood pressures causing ventricular enlargement as well as LV systolic and diastolic dysfunction [6]. Smoking-related micro-alterations would increase myocardial oxygen demand, making the heart more susceptible to microvascular ischemic damage, which could be related to LV abnormality [6, 32]. In addition, increased sympathetic stimulation via nicotine, carbon monoxide exposure, endothelial dysfunction, direct cellular damage, and oxidative stress were considered as factors contributing to several mechanisms of cardiovascular dysfunction [6, 33, 34]. As mentioned above, cigarette smoking could result in negative effects on cardiovascular function with several mechanisms. However, the present study could not detect the worse effect of smoking exposure against cardiac diastolic function. A previous study also reported an inconclusive association between smoking exposure and cardiac diastolic function owing to the difficulty in obtaining and measuring the data on diastolic function and the effect of other confounding factors [35]. The incomplete measurement of cardiac diastolic function and small sample size might make the results indefinite in the present study. Based on the discussion, although the present study included patients with relatively well-preserved cardiac function, cigarette smoking might have a worse impact on cardiac systolic function independent of respiratory function.

The study has several limitations. First, since this study was a single-center retrospective observational study, there is a risk of selection bias. The study also had a relatively small sample size that was adjusted for variables such as age, sex and respiratory function. Therefore, we were unable to investigate whether other factor would be associated with cardiac function. The participants’ background such as age and sex were heterogeneous among the three groups. It is well known that sex was directly associated with cardiac morphology including left and right ventricular volume and area [15]. Therefore, multiple linear regression analysis included age and male sex as well as respiratory parameters and lifetime pack-years. Furthermore, age in the multiple linear regression analysis might have been a confounding factor because the non-smokers were evidently older than those in the other groups, and their cardiac function was relatively better, regardless of their old age. Second, although the present study showed that ventricular diastolic dysfunction might not be affected by cumulative smoking exposure, several parameters regarding LV and RV diastolic function were unmeasured. Therefore, the results should be carefully interpreted considering the notable relationship between diastolic function and smoking exposure. Third, the present study excluded patients with a known history of ischemic heart disease; however, we could not completely eliminate the impact of atherosclerotic disease. Fourth, the patients’ medical records lacked information about alcohol and/or caffeine exposure which has the potential to affect the echocardiographic measurement results. Furthermore, it was difficult to accurately track and measure changes in the patients’ smoking status over a long period of time, which may have influenced the results. Fifth, LV and RV global longitudinal strain had about 10% of missing values and furthermore the study population was relatively small. Therefore, there might be discrepancies between the LVEF, right ventricular fractional area change and global longitudinal strain. Finally, the present study was observational in nature, and our findings that smoking exposure could have a negative impact on cardiac function independent of lung function need to be confirmed with a larger population-based cohort study.

## Conclusions

Cigarette smoking could be attributed to the worsening of systemic functions, especially cardiac and respiratory functions directly. In all models, the biventricular systolic function was mildly influenced by cumulative smoking exposure. Therefore, there was a negative effect of smoking exposure on the biventricular systolic function in patients with relatively well-preserved cardiac function independent of respiratory function.

## Supplementary information

**Additional file 1: Figure 1**. A representative of strain analyses using a semi-automated speckle tracking technique. The three apical views were used to obtain an average global peak systolic longitudinal strain with systole. Figures a, b and c are the apical long axis, 2-, 3- and 4-chamber views, respectively. Figure d shows the Bull’s eye plot of left ventricular global longitudinal strain assessed by speckle tracking technique.

**Additional file 2: Supplementary Table 1**. Spirometric parameters of the participants according to smoking exposure. **Supplementary Table 2**. Correlation among lifetime pack-years and spirometric parameters. **Supplementary Table 3**. Effect of lifetime pack-years on echocardiographic parameters.

## Data Availability

The datasets generated and/or analysed during the current study are not publicly available due the study dataset contains potentially identifying clinical information but are available from the corresponding author on reasonable request.

## Abbreviations

A: Late diastolic transmitral inflow peak velocity
ANOVA: Analysis of variance
a′: Mitral late diastolic annular peak velocity
a′t: Late diastolic peak velocity of the tricuspid annular velocity
COPD: Chronic obstructive pulmonary disease
E: Early diastolic transmitral inflow peak velocity
eGFR: Estimated glomerular filtration rate
e′: Mitral early diastolic annular peak velocity.
e′t: Early diastolic peak velocity of the tricuspid annular velocity
FEV1.0: Forced expiratory volume in one second
FVC: Forced vital capacity
LV: Left ventricular
LVEF: Left ventricular ejection fraction
LVN: Left ventricular mass
RV: Right ventricular
RVFAC: Right ventricular fractional area change
SD: Standard deviation
TAPSE: Tricuspid annular plane systolic excursion
VC: Vital capacity
