# Supplementary figures and images for: Influence of cigarette smoking on biventricular systolic function independent of respiratory function: a cross-sectional study
Source: BMC Cardiovasc Disord. 2020 Oct 15;20:451. doi: 10.1186/s12872-020-01732-6 (PMC7560055; doi:10.1186/s12872-020-01732-6)

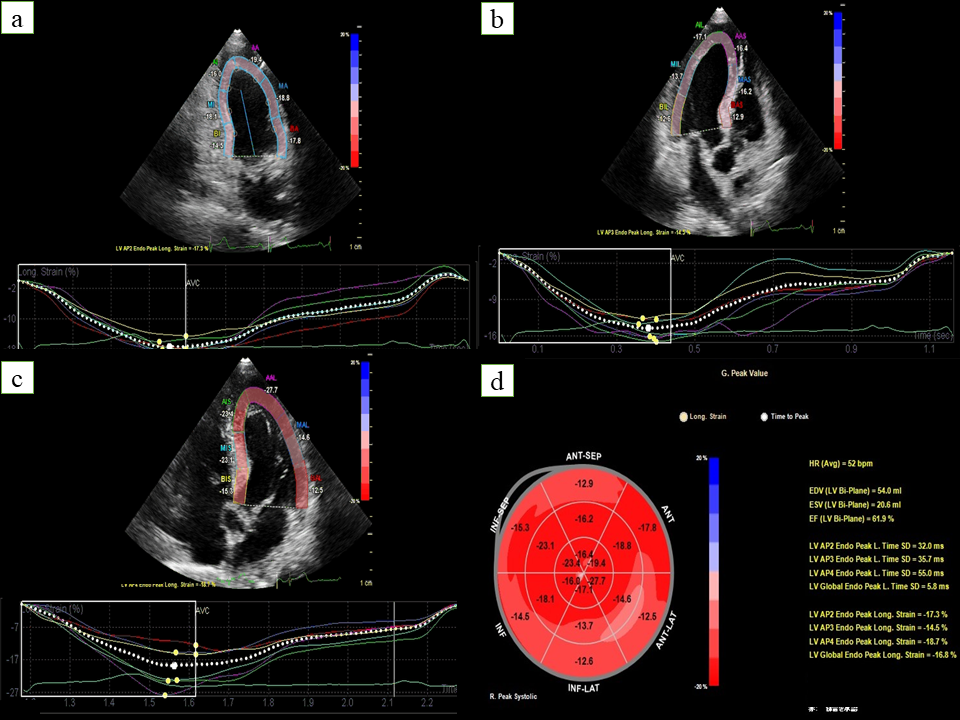

Supplement: Supplementary file 1 — Additional file 1: Figure 1. A representative of strain analyses using a semi-automated speckle tracking technique. The three apical views were used to obtain an average global peak systolic longitudinal strain with systole. Figures a, b and c are the apical long axis, 2-, 3- and 4-chamber views, respectively. Figure d shows the Bull’s eye plot of left ventricular global longitudinal strain assessed by speckle tracking technique. [file 12872_2020_1732_MOESM1_ESM.tif]
