# Supplementary material for: Influence of cigarette smoking on biventricular systolic function independent of respiratory function: a cross-sectional study
Source: BMC Cardiovasc Disord. 2020 Oct 15;20:451. doi: 10.1186/s12872-020-01732-6 (PMC7560055; doi:10.1186/s12872-020-01732-6)
Supplement: Supplementary file 2 — Additional file 2: Supplementary Table 1. Spirometric parameters of the participants according to smoking exposure. Supplementary Table 2. Correlation among lifetime pack-years and spirometric parameters. Supplementary Table 3. Effect of lifetime pack-years on echocardiographic parameters. [file 12872_2020_1732_MOESM2_ESM.docx]

**Supplementary Tables**

**Supplementary Table 1. Spirometric parameters of the participants according to smoking exposure.**

|  | **Cumulative smoking exposure** | | | |
| --- | --- | --- | --- | --- |
|  | **Non-Smoker**  **(n = 49)** | **Low exposure**  **(n = 40)** | **High exposure**  **(n = 95)** | ***p* value** |
| FEV 1.0, L | 1.84 ± 0.72 | 2.53 ± 0.77* | 2.38 ± 0.77* | <0.001 |
| FEV 1.0%, % | 79.9 (76.4 - 83.4) | 79.8 (76.1 - 83.6) | 77.9 (68.1 - 81.5)*† | 0.014 |
| %FEV1.0, % | 103.2 (87.8 - 117.5) | 94.8 (86.2 - 102.4) | 88.6 (78.1 - 98.7)* | <0.001 |
| FVC, L | 2.29 ± 0.89 | 3.18 ± 0.93* | 3.17 ± 0.83* | <0.001 |
| %FVC, % | 96.7 ± 22.3 | 101.1 ± 16.7 | 98.4 ± 18.2 | 0.56 |
| VC, L | 2.23 (1.88 - 2.56) | 3.28 (2.44 - 3.98)* | 3.25 (2.71 - 3.83)* | <0.001 |
| %VC, % | 92.5 (90.3 - 110.0) | 103.5 (91.3 - 114.9) | 104.2 (91.6 -112.4) | 0.69 |

Variables are indicated as mean ± SD for normally distributed variables and median [25^th^ – 75^th^ percentile] for non-normally distributed variables.

*p value < 0.05 for versus non-smoker, †p value < 0.05 for versus light smoker.

FEV: forced expiratory volume, FEV1.0: forced expiratory volume in one second, FEV1.0%: forced expiratory volume percentage in one second, %FEV1.0: percent predicted Forced Expiratory Volume in one second, FVC: forced vital capacity, %FVC: percent predicted forced vital capacity, VC: vital capacity, %VC: vital capacity percentage

**Supplementary Table 2.** **Correlation among lifetime pack-years and spirometric parameters.**

|  | **Correlation Coefficient** | ***p* value** |
| --- | --- | --- |
| Against lifetime pack-years |  |  |
| FEV 1.0, L | 0.176 | 0.017 |
| FEV 1.0%, % | -0.289 | <0.001 |
| %FEV1.0, % | -0.330 | <0.001 |
| FVC, L | 0.298 | <0.001 |
| %FVC, % | -0.030 | 0.69 |
| VC, L | 0.330 | <0.001 |
| %VC, % | 0.016 | 0.83 |
| Against %FVC |  |  |
| FEV 1.0, L | 0.637 | <0.001 |
| FEV 1.0%, % | -0.068 | 0.36 |
| %FEV1.0, % | 0.690 | <0.001 |

FEV1.0: forced expiratory volume in one second, FEV1.0%: forced expiratory volume percentage in one second, %FEV1.0: percent predicted Forced Expiratory Volume in one second, FVC: forced vital capacity, %FVC: forced vital capacity percentage, VC: vital capacity, %VC: vital capacity percentage.

**Supplementary Table 3. Effect of lifetime pack-years on echocardiographic parameters**

|  | **Model 1** | | **Model 2** | |
| --- | --- | --- | --- | --- |
|  | **βcoefficient** | ***p* value** | **βcoefficient** | ***p* value** |
| Left ventricular end-diastolic diameter, mm | -0.016 (-0.050, 0.017) | 0.34 | -0.014 (-0.049, 0.021) | 0.43 |
| Left ventricular end-systolic diameter, mm | 0.007 (-0.019, 0.033) | 0.61 | 0.006 (-0.021, 0.033) | 0.65 |
| Left ventricular end-diastolic volume index, ml/m^2^ | -0.057 (-0.144, 0.029) | 0.19 | -0.069 (-0.161, 0.022) | 0.14 |
| Left ventricular end-systolic volume index, ml/m^2^ | 0.004 (-0.049, 0.057) | 0.88 | 0.007 (-0.049, 0.062) | 0.82 |
| Left ventricular mass index, g/m^2^ | -0.046 (-0.179, 0.086) | 0.49 | -0.021 (-0.160, 0.118) | 0.77 |
| Left atrial volume index, ml/m^2^ | -0.047 (-0.126, 0.032) | 0.24 | -0.045 (-0.128, 0.039) | 0.30 |
| Aortic root diameter, mm | -0.019 (-0.044, 0.005) | 0.12 | -0.012 (-0.038, 0.013) | 0.35 |
| A wave velocity, cm/s | 0.020 (-0.105, 0.144) | 0.76 | 0.032 (-0.098, 0.163) | 0.63 |
| Right ventricular end-diastolic area index, cm^2^/m^2^ | 0.008 (-0.008, 0.024) | 0.32 | 0.007 (-0.010, 0.240) | 0.42 |
| Right ventricular end-systolic area index, cm^2^/m^2^ | 0.011 (0.0003, 0.021) | 0.044 | 0.010 (-0.001, 0.021) | 0.072 |
| Tricuspid valve annular diameter, mm | 0.028 (-0.005, 0.061) | 0.090 | 0.038 (0.004, 0.073) | 0.028 |
| Inferior vena cava, mm | -0.004(-0.025, 0.017) | 0.71 | -0.009 (-0.032, 0.014) | 0.46 |

Beta coefficients were interpreted as the absolute change in the dependent variable per change in the intensity of pack year.

Model 1 is adjusted for age and sex.

Model 2 is adjusted for Model 1 plus FEV1.0% and %FVC.

FEV1.0%: forced expiratory volume percentage in one second, %FVC: percent predicted forced vital capacity.
